# Improvement in serum lipids and liver morphology after supplementation of the diet with fish oil is more evident under regular feeding conditions than under high-fat or mixed diets in rats

**DOI:** 10.1186/s12944-020-01339-y

**Published:** 2020-07-06

**Authors:** Silvia Godea (Lupei), Diana Ciubotariu, Mihai Danciu, Raoul Vasile Lupușoru, Cristina Mihaela Ghiciuc, Irina Cernescu, Nicolae Gheţu, Mihai Lupei, Cătălina Elena Lupușoru

**Affiliations:** 1 Department of Pharmacology, Faculty of Medicine, Grigore T. Popa University of Medicine and Pharmacy, Iaşi, Romania; 2 Department of Pathology, Faculty of Medicine, Grigore T. Popa University of Medicine and Pharmacy, Iaşi, Romania; 3 Department of Pathophysiology, Faculty of Medicine, Grigore T. Popa University of Medicine and Pharmacy, Iaşi, Romania; 4 Department of Plastic Surgery, Regional Oncology Institute, Iaşi, Romania; 5 Department of Natural and Synthetic Polymers, Faculty of Chemical Engineering and Environment Protection, Gheorghe Asachi Technical University, Iaşi, Romania

**Keywords:** N-3 PUFA, Atherogenesis, Steatosis, Fish oil, Cholesterol, Triglycerides, Weight gain, Cardiovascular diseases, Rats, Dietary supplements

## Abstract

**Background:**

Dietary n^− 3^ polyunsaturated fatty acids (PUFAs) have a role in preventing cardiovascular and hepatic diseases. However, their effects might differ significantly depending on individual dietary patterns. The aim of the present study was to evaluate the effects of dietary supplementation with ω-3 fatty acids (FA), administered in different schedules, on hepatic and aortic histological structure, lipid profile, and body weight (BW) in male Wistar rats under standard (SD), high-fat diet (HFD) and mixed feeding conditions.

**Methods:**

PUFA treatment consisted of the administration of 50 mg/kg fish oil (FO) daily by oral gavage. HFD was obtained by adding a suspension of 4% cholesterol, thiouracil and cholic acid to the animals’ drinking water. The rats were maintained on the diets for 6 weeks, and different schedules of PUFA administration were used. At 14, 28, and 42 days, the morphology of liver and aortic samples and the levels of total cholesterol (TC), high-density lipoprotein cholesterol (HDL), low-density lipoprotein cholesterol (LDL), and triglycerides (TG) were assessed.

**Results:**

The HFD groups exhibited significant hyperlipidemia and aortic inflammation, with progression to atherogenesis after 6 weeks. Administration of PUFAs slightly attenuated the aortic changes in these groups and reduced the liver’s tendency to steatosis. FO-induced metabolic improvement was more evident in SD than in HFD rats. For instance, after the first 2 weeks, SD animals that received PUFAs had significantly increased HDL levels vs. controls (62.375 ± 4.10 vs. 52.625 ± 8.38 mg/dL, *P <* 0.05), but HFD rats did not, and decreased TG levels were observed exclusively in the SD rats (57.6 ± 4.09 vs. 66 ± 4.69 mg/dL, *P <* 0.05). After 6 weeks of n^− 3^ PUFA administration, LDL was significantly lower in the SD rats than in controls (13.67 ± 4.13 vs. 30.83 ± 2.86 mg/dL, *P <* 0.001), but the decrease in the HFD rats, although significant (49.17 ± 5.85 mg/dL vs. 57.17 ± 4.96 g/dL, *P <* 0.05), was not as marked. In the mixed-diet groups, administration of 50 mg/kg/day FO for 14 days under SD conditions following 4 weeks of HFD slightly decreased TG (86.625 ± 11.67 vs. 73 ± 4.52 mg/dL, *P <* 0.05) and increased HDL (45.875 ± 5.28 vs. 56 ± 3.16 mg/dL). However, in these animals, n-3 PUFA administration had no effect on LDL or TC. Administration of half of the above dose failed to improve any biochemical parameters. FO protected against excessive weight gain mainly under SD conditions.

**Conclusions:**

The results show that FO confers more protection against cardiovascular risk factors (increased LDL and TG, decreased HDL) and liver lipid accumulation when given to rats consuming regular diets than when given to rats consuming a high-fat diet. This argues that priority should be given to consumption of a healthy diet rather than to the use of supplements. The effectiveness of n-3 PUFAs might be reduced in the case of hyperlipidic intake or after consumption of a high-fat diet.

## Introduction

Dietary supplementation with polyunsaturated fatty acids (PUFAs), mainly in the form of fish oil (FO), is known to have a protective and alleviating role in cardiovascular disease (CVD) and hepatic steatosis; improving the serum lipid profile and preventing the development of atherosclerosis are considered essential aspects of this role. High fat intake and disorders of lipid metabolism such as elevated serum total cholesterol (TC), low-density lipoprotein cholesterol (LDL) and triglycerides (TG), as well as decreased high-density lipoprotein cholesterol (HDL), are just some of the most frequent and important risk factors associated with CVD. High fat intake is also associated with hepatic lipid accumulation and increased TG storage, which further lead to steatosis, triggering metabolic dysfunctions such as insulin resistance and dyslipidemia [1]. Currently, there is increased awareness not only among scientists but also among the general population of the negative effects of a fat-rich diet and its propensity to cause atherosclerosis, CVD, fatty liver disease, and stroke [2, 3].

Despite their negative role in the pathogenesis of many diseases, fats are essential for living organisms. PUFAs represent a category of essential fats that can be classified according to the position of the first double bond relative to the end methyl group. N-3 and n-6 fatty acids (FA) are the most biologically significant classes of PUFAs and are synthesized from the essential FAs alpha-linolenic and linoleic acid, respectively. N-3 PUFA intake is associated with a protective effect against CVD. The n-6:n-3 ratio is very important to human health, as n-3 and n-6 PUFAs compete with one another for the active site of the enzyme responsible for their metabolism. An overabundance of one class will limit the metabolic production of the longer- chain products of the other; thus, decreasing the n-6:n-3 ratio facilitates the metabolism of alpha-linolenic acid. The typical Western diet provides n-6 and n-3 PUFAs in ratios ranging from 8:1 to 25:1 [4], in severe contrast to the ratio of approximately 4:1 recommended by health agencies [5]. Several animal studies have shown the inhibitory effect of n-3 PUFA administration on the development and destabilization of atherosclerotic plaques [6–9]; randomized human trials showed that diets high in n-3 PUFAs reduce CVD mortality endpoints such as sudden death and fatal myocardial infarction [10–13]. N-3 PUFAs have also been shown to protect against hepatic steatosis [14, 15]. Fish products, which typically contain large amounts of ω-3 FAs such as eicosapentaenoic acid (EPA) and docosahexaenoic acid (DHA), are considered the most common dietary source of n-3 PUFAs [16].

Since the 1970s, increased adherence to the Western diet, which is rich in hydrogenated FAs, has been observed, mostly among young individuals. In the United States of America in recent decades, there has been a transition from diets high in complex carbohydrates and fiber to diets rich in sugars, fats, and animal products [17]. However, more recently, increased awareness in the Western population of the role of a healthy diet in preventing metabolic diseases and a tendency to reduce saturated FA intake was observed. Eighty-three percent of EU citizens consider that they have healthy diets, with a peak in the Netherlands (95%), and 22% of respondents report that they have changed their eating habits during the past year and are now consuming more vegetables, less fat and sugar and fewer calories and drinking more water [18]. Even in the United States of America, national estimates report that obesity has leveled off at a prevalence of approximately 35% during the last 12 years [19]. Mediterranean diets based on PUFAs such as ω-3 and ω-6 have become ever more popular [20], as has the use of ω-3 FA supplements such as FO. The global ω-3 market was estimated at 2.29 billion USD in 2018 [21]. Numerous studies have shown the benefits of ω-3 FAs, but the data are sometimes contradictory, and the molecular mechanisms involved in atherosclerosis and liver steatosis prevention are poorly understood. Conclusive evidence has yet to be provided for the presumed anti-atherogenic action of ω-3 FAs. There might be significant differences in the impact of PUFAs on the mentioned processes due to individuals’ dietary particularities. A large segment of the population does not exclusively follow only one type of diet [22]. The action of n-3 PUFAs can be influenced by association with other substances or by an individual’s metabolic profile. PUFA sources and EPA:DHA ratios are extremely variable, and this can cause differences in results. Therefore, a study designed to analyze, compare and quantify the efficacy of dietary ω-3 FA supplementation under conditions of Western, Mediterranean, and heterogeneous diets or simulation of different metabolic profiles would bring new insight into this matter.

The main purpose of this study was to evaluate the effects of dietary n-3 PUFA supplementation on body weight (BW) and on the main parameters of lipid metabolism (cholesterol, TG, and other parameters) in an experimental rat model using different diets. In this study, known atherogenic factors (cholesterol, cholic acid and 2-thiouracil) and potential anti-atherogenic factors (FO) were administered successively or simultaneously. The aim was to quantify early and persistent biological responses to n-3 PUFA administration in animals with induced hyperlipidemia and to correlate these responses with histological aortic and hepatic modifications (atherogenesis, steatosis) before or after changing the animals’ diets.

## Methodology

### Ethical policies

This study complied with the European Guidelines for Human and Animal Rights, Directive 2010/63/EU, regarding the protection of animals used for scientific purposes and was approved by the Research Ethics Committee of the Grigore T. Popa University of Medicine and Pharmacy in Iaşi, Romania (registration no. 25812/11.XI.2014).

### Laboratory animals

Sixty five-week-old male albino Wistar rats (nonconsanguineous) were used in the experiments. The animals weighed 150 ± 10 g each at the start of the experiment and were purchased from the I. C. Cantacuzino National Institute of Research and Development for Microbiology and Immunology, Bucharest, Romania. The animals were individually housed in polypropylene cages according to European standards in the Laboratory for Pharmacology Research in a controlled environment (temperature 23 ± 2 °C, 50–60% relative humidity, central ventilation and artificial light-dark cycles of 12 h/12 h). The animals had free access to water and standard food, which were provided ad libitum.

The rats were acclimatized for 7 days prior to the beginning of the experimental study.

### Substances

Cholesterol (95%) was purchased from Alfa Aesar (Heysham, UK), cholic acid was obtained from Merck (Darmstadt, Germany), and 2-thiouracil and concentrated n-3 fatty acids (DHA/EPA, 200/250 mg) were obtained from Sigma-Aldrich Co. (Deisenhofen, Germany). All other chemicals and reagents (analytical grade) were purchased from Alfa Aesar (Heysham, UK).

### Diets

The standard diet (SD) consisted of pellets manufactured by the I. C. Cantacuzino National Institute of Research and Development for Microbiology and Immunology, Bucharest, Romania. The nutritional composition of the pellets was as follows: crude protein 18.55%, crude fat 2.65%, crude fiber 6.8%, salts 0.48%, and water 10.65%. The SD was free of EPA and DHA.

The hypercholesterolemia diet (high-fat diet, HFD) consisted of the standard diet plus supplementation of the drinking water with a standardized suspension (CCT suspension) containing 4% cholesterol, 1% cholic acid and 0.5% 2-thiouracil. The diet was designed to induce atherogenic changes and hepatic steatosis as well as disturbances of lipid metabolism (hyperlipidemia), as suggested by similar studies [23–26].

The n-3 PUFA treatment consisted of oral gavage with a suspension containing FO obtained from capsules (50 mg/kg BW daily) [12, 27].

### Experimental design and diet administration

The rats were randomly divided into 6 groups of 10, and the experiment was conducted for 42 days.

The first group (**SD, standard diet**) was used as a control. Food was provided as a standard diet with no supplementation during the entire 42 days of the experiment.

The second group (**n3**) received standard food and FO (50 mg/kg/day) for 42 days. The treatment received by this group was designed to assess the benefits of dietary supplementation with ω-3 FA under regular dietary conditions.

The third group (**HFD**) received standard food plus the CCT suspension in the drinking water, as described in the “Diets” section, for 42 days. This group served as a model of HFD-triggered disease.

The fourth group (**HFD + n3**) received drinking water containing the CCT suspension as well as FO (50 mg/kg/day) for 42 days. This group was used to assess the benefits of dietary ω-3 FA under HFD conditions.

The fifth group (**CA1**: combined alternative 1) received the HFD and n-3 PUFAs for 14 days followed by HFD only from days 15–28; during the last 2 weeks of the experiment (days 29–42), the animals in this group received the SD and 50 mg/kg/day n-3 PUFAs.

The sixth group (**CA2**: combined alternative 2) received HFD with no FO for the first 14 days, both n-3 PUFAs and the HFD from days 15–28; during the last 2 weeks of the experiment (days 29–42), the animals in this group received the SD and 25 mg/kg/day n-3 PUFAs (Table 1).

**Table 1:** Diet administration schedule for each study group (DW – distilled water with no CCT suspension; CCT – standardized suspension containing 4% cholesterol, 1% cholic acid, and 0.5% 2-thiouracil; n-3 PUFA – oral gavage of a suspension containing fish oil from capsules, 50 mg/kg/day; the dose was reduced to 25 mg/kg/day in the CA2 group on days 29–42)

Feeding cycle	SD	n3	HFD	HFD + n3	CA1	CA2
**days 1–14**	DW	n3 PUFA	CCT	CCT + n3 PUFA	CCT + n3 PUFA	CCT
**days 15–28**	DW	n3 PUFA	CCT	CCT + n3 PUFA	CCT	CCT + n3 PUFA
**days 29–42**	DW	n3 PUFA	CCT	CCT + n3 PUFA	n3 PUFA	n3 PUFA, half dose

The CA1 and CA2 groups served as models of different transitions from HFD to a regular diet that more closely resembles the Mediterranean diet, using n-3 PUFAs for cardiovascular protection.

To reproduce the stress conditions induced by oral gavage, distilled water was administered by gavage to each animal that did not receive FO gavage on that day.

During the experiment, BW, the quantities of food and water consumed and the amounts of urine and feces produced were measured daily for each animal.

After each 14-day feeding cycle, two rats from each group were euthanized by intraperitoneal injection of sodium pentobarbital (45 mg/kg) to minimize tissue alterations and permit better histological examination. At the end of the 42-day experiment, all of the remaining rats were euthanized under the same conditions for histological assessment of liver and aorta samples.

### Biochemical determination

Total cholesterol (TC), low-density lipoprotein cholesterol (LDL) and high-density lipoprotein cholesterol (HDL) levels were determined using kits supplied by BD Biosciences Co. (Heidelberg, Germany) and a VITROS® 350 Chemistry System biochemical analyzer (Johnson & Johnson Ortho-Clinical Diagnostics) (colorimetric methods). Blood samples were collected from the orbital sinus on days 14, 28 and 42 and stored in standard vacutainers. Values are expressed in mg/dL.

### Histopathological examination

After each rat was euthanized, its entire aorta and liver were removed, rinsed with cold saline solution, dried with filter paper, grossly examined under a magnifying glass (5x) and routinely processed. Six to eight 4-mm samples were taken from different levels of each aorta and cut into transverse and longitudinal sections. Fixation was performed using 10% neutral buffered formalin at room temperature for at least 24 h. The specimens were then dehydrated in increasing concentrations of ethanol (75 to 99.6%), immersed in xylene and paraffin embedded. Sections 4–5 μm in thickness were stained with hematoxylin and eosin (HE) and van Gieson’s trichrome for light microscopic evaluation. Examination of the aortic samples was performed in longitudinal and transverse sections and consisted of counting the collagen layers. In evaluation of the liver samples, the study focused on the semiquantitative evaluation of fatty change.

### Statistical data analysis

Statistical analysis was performed using SPSS software version 20 for Windows (IBM, Chicago, Illinois, United States of America). Data were first checked for normality in each group and for each tested parameter using the Shapiro-Wilk test. When the data showed a normal distribution, the results were expressed in graphs and tables as average ± standard deviation (e.g., quantitative data values for body weight, TC, LDL cholesterol, HDL cholesterol, and TG). In such cases, one-way ANOVA was used to test for differences among groups; the paired samples t-test was used for same-group comparisons at different time points. When the data were not normally distributed, the Kruskal-Wallis test was used for between-groups comparison; these data are presented in the tables as median and quartiles (q1 – q3). *P* < 0.05 was considered significant.

## Results

### Body weight, growth, food and water intake

During the acclimatization period and the 42 days thereafter, the BW of the animals in all 6 groups increased constantly regardless of their diets. At the end of the acclimatization period, the percentage increase in BW was 25.69 ± 4.75% vs. 7 days previously in the SD group and similar in the other 5 groups. The last of the 7 acclimatization days was considered day 0.

**Fig. 1:**
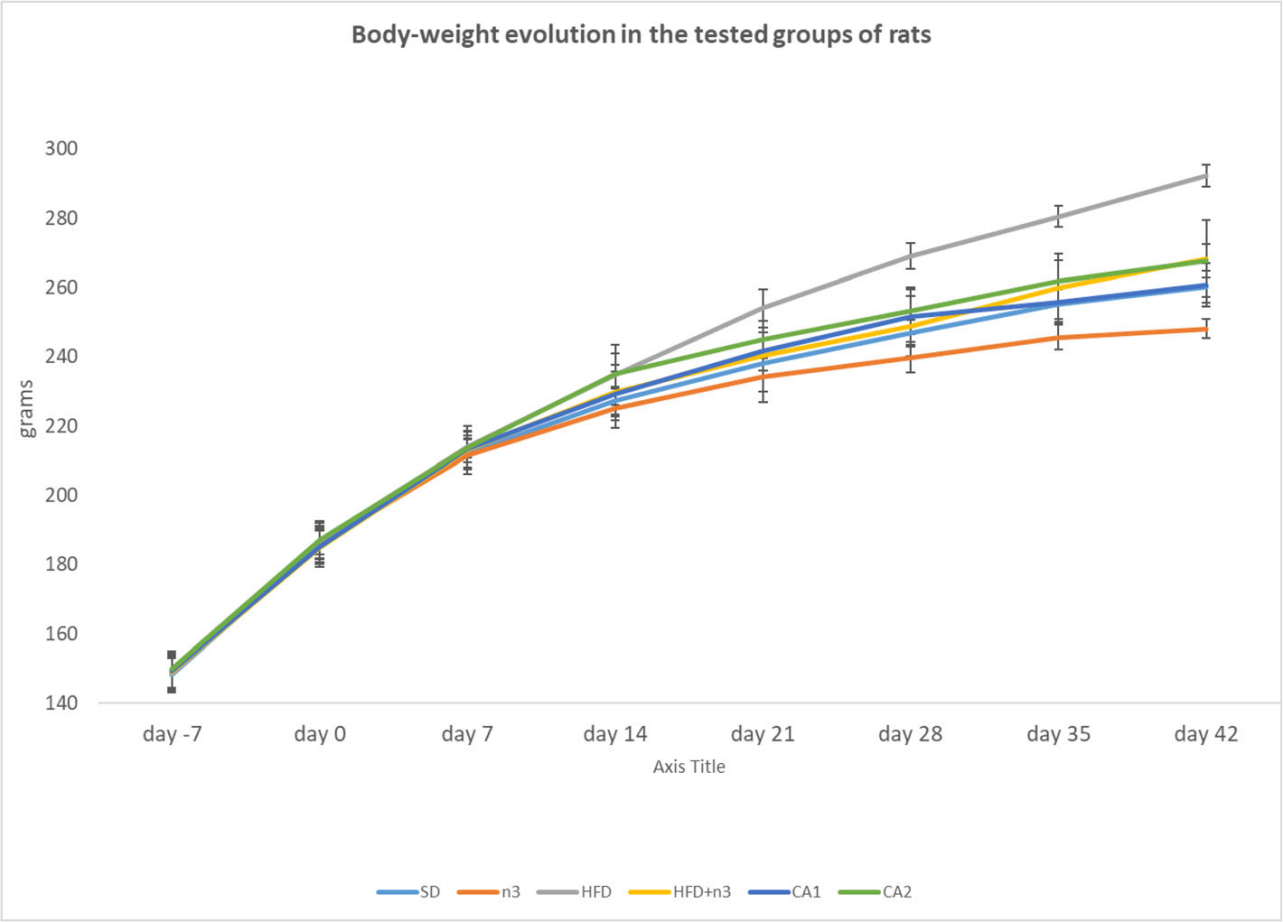
Evolution of body weight during the acclimatization period and during the 6 weeks of different dietary regimes (in grams, average ± standard deviation)

At the end of 6 weeks on the different dietary regimes following acclimatization, the HFD rats showed the greatest increase in BW (from 187.1 ± 5.86 g on day 0 to 292.33 ± 3.26 g on day 42); the smallest increase was observed in the n3 group (from 186.1 ± 5.86 g on day 0 to 248.17 ± 2.85 g on day 42). In the SD group, BW increased from 186.1 ± 4.25 g on day 0 to 260.67 ± 4.58 g on day 42. In the HFD + n3 group, it increased from 185.1 ± 4.15 g on day 0 to 268.33 ± 11.15 g on day 42, indicating that n-3 PUFAs offer significant protection against excessive weight gain (WG) under HFD conditions (*P <* 0.001, HFD vs. HFD + n3 groups). In the CA1 group, the BW increase was higher than that observed in the n3 group (*P <* 0.01) and lower than that observed in the HFD group (*P <* 0.001); for the CA2 group, it was higher than that in the n3 group (*P <* 0.01) and lower than that in the HFD group (*P <* 0.01). The intermediate WG in the CA1 and CA2 groups (between those of the HFD and n3 groups) can be explained by the addition of n-3 PUFAs during half of the HFD period and mainly during the last 14 days under SD conditions. The evolution of BW during the experiment is illustrated in Fig. 1.

To evaluate BW increase at different stages of the experiment, BW increase was calculated as the difference in the animal’s weight on the day of testing vs. 7 days previously (i.e., day 28 vs. 21), considering the first day after acclimatization as day 1. The data for each of the 6 weeks and for the 42 days during which the animals followed the dietary regimes are summarized in Table 2.

**Table 2:** Percentage (%) WG in groups exposed to different diets. The weekly WG (BW percentage increase vs. 7 days previously) and the BW percentage increase during the 6 weeks of the dietary regime are shown. For the first week (day 7 vs. day 0), data are expressed as median and quartiles (q1-q3) because the data are not normally distributed; the data for the following five weeks are expressed as average ± standard deviation because the values for percentage increase in BW showed a normal distribution. The last line presents the percentage BW increase over the 6 weeks of different dietary regimes in the studied groups (average ± standard deviation)

	SD	n3	HFD	HFD + n3	CA1	CA2
day 7 vs. day 0	14.02 (13.83–14.53)	14.02 (13.61–16.02)	13.98 (13.72–19.57)	14.57 (13.61–20.11)	14.02 (13.83–21.35)	13.94 (13.61–17.55)
day 14 vs. day 7	7.17 ± 0.57	6.29 ± 1.00 ↓ vs. SD	9.73 ± 3.54 ↑ vs. SD ↑↑ vs. n3	7.72 ± 3.70	7.39 ± 2.43	10.03 ± 3.72 ↑ vs. SD ↑↑ vs. n3
day 21 vs. day 14	4.90 ± 0.18	3.59 ± 1.31 ↓ vs. SD	8.03 ± 3.82 ↑ vs. SD ↑↑ vs. n3	4.10 ± 1.05 ↓ vs. HFD	5.83 ± 2.26	4.23 ± 2.41 ↓ vs. HFD
day 28 vs. day 21	3.68 ± 0.20	2.44 ± 1.61 ↓ vs. SD	5.94 ± 2.00 ↑↑ vs. SD ↑↑ vs. n3	3.62 ± 1.31 ↓ vs. HFD	4.14 ± 2.32	3.36 ± 1.10 ↓↓ vs. HFD
day 35 vs. day 28	3.31 ± 0.19	2.72 ± 0.51 ↓ vs. SD	4.28 ± 0.71 ↑↑ vs. SD ↑↑ vs. n3	3.30 ± 0.78 ↓ vs. HFD	2.83 ± 1.06 ↓ vs. HFD	3.03 ± 1.42
day 42 vs. day 35	1.96 ± 0.49	1.02 ± 0.51 ↓↓ vs. SD ↓↓ vs. SD	4.22 ± 0.53 ↑↑↑ vs. SD ↑↑↑ vs. n3	3.26 ± 1.21 ↑ vs. SD ↑↑ vs. n3	1.89 ± 0.84 ↓↓↓ vs. HFD ↓ vs. HFD + n3	2.24 ± 1.25 ↓↓ vs. HFD
day 42 vs. day 0	28.70 ± 0.97%	25.39 ± 1.67% *P <* 0.01 vs. SD ↓↓ vs. SD	36.08 ± 1.92% *P <* 0.001 vs. n3 *P <* 0.001 vs. SD	30.33 ± 2.06% *P <* 0.01 vs. n3	29.39 ± 2.07% *P <* 0.01 vs. n3 *P <* 0.001 vs. HFD	30.68 ± 2.30% *P <* 0.01 vs. n3 *P <* 0.01 vs. HFD

Legend: ↓ - decrease, 0.01 < *P <* 0.05; ↓↓ - decrease, 0.001 < *P <* 0.01; ↓↓↓ - decrease, *P <* 0.001;

↑ - increase, 0.01 < *P <* 0.05; ↑↑ - increase, 0.001 < *P <* 0.01; ↑↑↑, − increase, *P <* 0.001.

There were no significant differences among the groups on day 7.

From day 7 to day 14, a slight difference in BW was observed: WG was more evident (*P <* 0.05) in the HFD group than in the SD group and less evident in the n3 group than in the SD group (*P <* 0.05); addition of n-3 PUFAs to the HFD did not result in significant protection against increased WG in the HFD + n3 or CA1 groups.

The tendency of the HFD group to gain more weight than the SD group and the tendency of the n3 group to gain less weight than the SD group was also evident in the subsequent 4 weeks of the study. Addition of FO to the HFD offered a relative protective effect against excessive WG in the HFD + n3 group compared to the HFD group during weeks 3, 4 and 5 of the experiment but not during the last week.

Between days 14 and 21, the CA2 group, which during that period received the HFD with n-3 PUFA supplementation, exhibited relative protection against excessive WG (*P* < 0.05 vs. HFD), and the protection continued from day 21 to day 28 (*P* < 0.01, CA2 vs. HFD).

Addition of PUFAs to the SD on days 28 through 35 or on days 35 through 42 in the CA1 and CA2 groups, both of which had previously received the high-fat diet, did not reduce BW gain compared to the SD group. When administered on days 35 through 42, however, FO offered relative protection against excessive WG in the CA1 and CA2 groups.

No significant changes in the amounts of food and water consumed or in the amounts of urine and feces excreted by the animals were observed at any of the experimental time points; thus, no correlation of these parameters with BW could be established. No relevant changes in feeding pattern were seen in the CA1 or CA2 groups when their diets were changed.

### Biochemical determinations

*Total serum cholesterol* was initially similar in the studied groups, with values ranging from 78 to 103 mg/dL (Table 3).

**Table 3:** Serum concentrations of total cholesterol (TC), LDL-cholesterol, HDL-cholesterol and triglycerides in the studied groups of rats (mg/dL), average ± standard deviation after 14, 28 and 42 days of different diets/protocols

		SD	n3	HFD	HFD + n3	CA1	CA2
TC	initially	87.8 ± 5.43	88.6 ± 6.17	89 ± 5.62	88.4 ± 8.47	88.1 ± 5.82	87.6 ± 5.60
	14 days	90.9 ± 6.13	83.9 ± 4.12↓ (a)	111.8 ± 8.35↑↑↑ (b)(b)(b)	97.9 ± 6.45↑ (b) (−)(−)(−)	97.8 ± 6.64↑ (b) (−)(−)(−)	114.7 ± 8.62↑↑↑ (b)(b)(b)
	28 days	93.375 ± 6.48	82.625 ± 4.89 (a)(a)	137.375 ± 12.73↑↑(b)(b)(b)	100.375 ± 7.61↑ (b) (−)(−)(−)	111.5 ± 8.67↑↑↑ (b)(b)(b) (−)	111.75 ± 8.71 (b)(b)(b) (−)(−)(−)
	42 days	94 ± 6.90	81.83 ± 5.91 (a)(a)	151.167 ± 10.09↑↑ (b)(b)(b)	115.667 ± 6.15↑↑ (b)(b) (−)(−)(−)	103.167 ± 7.68 (−)	110.167 ± 8.06 (b)(b) (−)(−)(−)
HDL-CT	initially	50.5 ± 4.01	51.6 ± 4.76	50.9 ± 4.98	51.9 ± 4.15	52.7 ± 4.88	49.1 ± 5.67
	14 days	49.8 ± 3.85	55.7 ± 5.51↑ (b)	49.7 ± 5.62	52.4 ± 6.82	52.8 ± 7.35	48.8 ± 5.57
	28 days	52.625 ± 8.38	61.625 ± 3.89↑ (b)	41.75 ± 6.16↓ (a)	50.375 ± 5.58 (b) (+)	45.875 ± 5.28 (b)(b)	47.625 ± 6.59 (b)(b)
	42 days	50.5 ± 6.77	63.83 ± 4.45 (b)(b)	40.33 ± 4.32 (a)	47.83 ± 4.71 (b) (+)	56 ± 3.16↑ (+)(+)(+)	50.67 ± 5.61 (b) (+)(+)
LDL-CT	initially	27.3 ± 4.83	27.2 ± 4.28	26.7 ± 5.83	26.2 ± 7.05	26.4 ± 6.33	25.7 ± 2.83
	14 days	28.1 ± 3.10	24.1 ± 3.31↓ (a)	34.4 ± 3.10↑↑↑ (b)(b)(b)	31.2 ± 3.33↑ (b) (−)	31.2 ± 2.58↑ (b) (−)	33.8 ± 2.90↑↑↑ (b)(b)(b)
	28 days	27 ± 3.55	16.875 ± 5.38↓↓ (a)(a)(a)	45.625 ± 4.90↑↑ (b)(b)(b)	38 ± 4.14↑↑ (b)(b)(b) (−)(−)	42 ± 4.57↑↑↑ (b)(b)(b)	41.125 ± 2.69↑↑↑ (b)(b)(b) (−)
	42 days	30.83 ± 2.86	13.67 ± 4.13 (a)(a)(a)	57.17 ± 4.96↑↑↑ (b)(b)(b)	49.17 ± 5.85↑ (b)(b)(b) (−)	36.67 ± 5.28 (b) (−)(−)(−)	40.33 ± 2.50 (b)(b)(b) (−)(−)(−)
TG	initially	64.8 ± 8.63	63.5 ± 7.37	65.6 ± 5.95	62.9 ± 3.96	62.7 ± 5.44	66.6 ± 4.43
	14 days	66 ± 8.69	57.6 ± 4.09↓ (a)	75.4 ± 5.48↑↑ (b)(b)	70.9 ± 5.30↑↑	71.7 ± 5.62↑↑	75.6 ± 5.67↑↑ (b)(b)
	28 days	67 ± 10.4	55.375 ± 6.05 (a)	90.625 ± 6.02↑↑↑ (b)(b)(b)	83.625 ± 6.82↑↑↑ (b)(b) (−)	86.625 ± 11.67↑↑↑ (b)(b)	85 ± 9.40↑↑ (b)(b)(b) (−)(−)(−)
	42 days	65 ± 10.16	49.33 ± 6.4 (a)(a)	104 ± 7.56↑↑ (b)(b)(b)	91 ± 8.56 (b)(b)(b) (−)	73 ± 4.52↓ (−)(−)(−)	83.33 ± 7.47 (b)(b) (−)(−)(−)

Legend:

**vs. 2 weeks previously**: ↓ - decrease, 0.01 < *P <* 0.05; ↓↓ - decrease, 0.001 < *P <* 0.01; ↑ - increase, 0.01 < *P <* 0.05; ↑↑ - increase, 0.001 < *P <* 0.01; ↑↑↑ - increase, *P <* 0.001;

**vs. SD group**: (a) - decrease, *P <* 0.05; (a)(a) - decrease, 0.001 < *P <* 0.01; (a)(a)(a) - decrease, *P <* 0.001; (b) - increase, *P <* 0.05; (b)(b) - increase, 0.001 < *P <* 0.01; (b)(b)(b) - increase, *P <* 0.001;

For the HFD + n3, CA1, and CA2 groups, the ***P*values vs. the HFD group** are shown: (−) - decrease, 0.01 < *P <* 0.05; (−)(−) - decrease, 0.001 < *P <* 0.01; (−)(−)(−) - decrease, *P <* 0.001; (+) - increase, 0.01 < *P <* 0.05; (+)(+) - increase, 0.001 < *P <* 0.01; (+)(+)(+) - increase, *P <* 0.001.

At the end of the first 2 weeks, TC in the n3 rats was lower than initially observed (*P <* 0.05) and lower than that in the SD rats (*P <* 0.05). In the HFD and CA2 groups, TC was higher at 2 weeks than it had been initially and higher than that in the SD rats (*P <* 0.001). In the CA1 and HFD + n3 rats, PUFAs offered relative protection against increased TC (*P <* 0.001 vs. the HFD and CA2 groups), but TC was higher vs. initially (*P <* 0.05) and vs. the n3 and SD groups (*P <* 0.001 and 0.01 < *P <* 0.05, respectively).

Four weeks into the experiment, the HFD rats continued to show increasing TC levels (*P <* 0.001 vs. 14 days previously). In the n3 group, TC was unchanged vs. 14 days previously but significantly lower than in the SD rats (*P <* 0.01). In the HFD + n3 group, TC was insignificantly higher vs. 2 weeks previously but lower than in the HFD group (*P <* 0.001). In the HFD + n3 group, TC was lower than in the HFD group (*P <* 0.001) but higher than the initial value and higher than the level in the SD group (*P <* 0.05); it was also higher than the level in the n3 group (*P <* 0.001). In the CA1 and CA2 groups, TC concentrations were higher (*P <* 0.05) than those in the HFD + n3 group yet much lower (*P <* 0.001) than those in the HFD group.

After 6 weeks, there was a continuation of the increasing trend of TC in the HFD group (*P <* 0.01 vs. 14 days previously). In the n3 group, TC was insignificantly changed vs. 14 and 28 days previously. In the HFD + n3 group, TC was lower than in the HFD group (*P <* 0.001) but higher than observed 2 weeks previously (*P <* 0.05). In the CA1 and CA2 groups, TC was insignificantly lower than it had been 14 days previously, indicating that switching to the standard diet after 4 weeks on the HFD has a significant TC-lowering effect only after at least 2 weeks, even under PUFA-protection conditions.

*Serum HDL cholesterol* was initially similar in the studied groups, with values ranging from 41 to 64 mg/dL.

After 14 days, there were no significant changes in HDL levels in the SD, HFD, HFD + n3, CA1 and CA2 groups. In the n3 group, HDL was higher than it had been initially and higher than in the SD, HFD and CA2 groups (*P <* 0.05) but insignificantly different vs. the HFD + n3 and CA1 groups.

After 4 weeks, HDL was higher in the n3 rats than in the SD group and higher than it had been 2 weeks previously (*P <* 0.05) and initially (*P <* 0.01). In the HFD rats, HDL was lower than in the SD group and lower than it had been 14 days previously (*P <* 0.05) and initially (*P <* 0.01). At that point in the experiment, the protective role of a normal diet combined with FO vs. an HFD with no PUFA protection was more evident than it had been 14 days previously (n3 vs. HFD: *P <* 0.001) in terms of HDL increase. In the HFD + n3 rats, HDL was insignificantly modified compared to 14 and 28 days previously, but it was higher vs. the HFD group (*P <* 0.05) and lower vs. the n3 group (*P <* 0.001). In the CA1 rats, HDL was insignificantly lower vs. the HFD + n3 group and vs. 2 weeks previously but lower vs. initially (*P <* 0.01). In the CA2 group, serum HDL was insignificantly changed vs. initially and vs. the HFD, HFD + n3, and CA1 groups, but it was significantly lower than the level in the n3 group (*P <* 0.001).

On day 42, in the n3 group, HDL was higher vs. the SD group (*P <* 0.01). Also, it was higher vs. initially and vs. 4 weeks previously (*P <* 0.01), but insignificantly higher than it had been 2 weeks previously. In the HFD group, the HDL value confirmed the decreasing trend in this parameter; HDL was lower than in the SD group (*P <* 0.05) and lower than it had been initially and 28 days previously (*P <* 0.01) but not lower than it had been 14 days previously. In the HFD + n3 group, HDL was insignificantly modified vs. 14 and 28 days previously, significantly higher vs. the HFD group (*P <* 0.05), and significantly lower vs. the n3 group (*P <* 0.001) and vs. initially (*P <* 0.05). In the CA1 group, HDL was higher vs. the HFD + n3 group (*P <* 0.01) and vs. the HFD group (*P <* 0.001), higher than it had been 2 weeks and 4 weeks previously (*P <* 0.05), and insignificantly modified vs. initially. In the CA2 group, HDL was insignificantly changed vs. initially, vs. 2 and 4 weeks previously, and vs. the CA1 group, but it was lower than in the n3 group (*P <* 0.01) and higher than in the HFD group (*P <* 0.01).

*Serum LDL cholesterol* was initially similar in the studied groups, with values ranging from 15 to 39 mg/dL.

After the first 2 weeks, the n3 rats had lower LDL vs. initially and vs. the SD rats (*P <* 0.05). In the HFD and CA2 groups, LDL was higher vs. initially and vs. the SD rats (*P <* 0.001). In the CA1 and HFD + n3 groups, administration of n-3 PUFAs conferred some protection against increased LDL levels: LDL was lower (*P <* 0.05) vs. the HFD and CA2 groups yet higher vs. initially and vs. the SD (*P <* 0.05) and n3 groups (*P <* 0.001).

At 28 days, the HFD rats continued to show increasing LDL levels (*P <* 0.01 vs. 14 days previously). In the n3 rats, LDL was lower vs. 14 days previously (*P <* 0.01) and vs. the SD rats (*P <* 0.001). In the HFD + n3 group, LDL was higher vs. 2 weeks previously (*P <* 0.001), vs. initially (*P <* 0.01) and vs. the SD and n3 groups (*P <* 0.001) but lower vs. the HFD group (*P <* 0.01). Thus, compared to 14 days previously, the increase in LDL was greater in the HFD + n3 group than in the HFD group. In the CA1 and CA2 groups, LDL was insignificantly higher vs. the HFD + n3 group and insignificantly lower vs. the HFD group.

Six weeks into the study, there was a slight yet significant increase in LDL compared to the initial level in the SD group (*P <* 0.05). In the HFD group, there was a continuation of the upward trend in LDL concentration (*P <* 0.01 vs. 14 days previously). In the n3 rats, LDL was insignificantly changed compared to 14 days previously yet significantly higher vs. initially (*P <* 0.01), vs. 28 days previously (*P <* 0.05) and vs. the control group (*P <* 0.001). In the HFD + n3 group, LDL was lower than it was in the HFD group (*P <* 0.05) and higher than it had been 2 weeks previously (*P <* 0.05). In the CA1 and CA2 groups, LDL was insignificantly lower vs. 14 days previously but lower vs. the HFD + n3 group (*P <* 0.01) and vs. the HFD group (*P <* 0.001).

**Serum triglycerides** were initially similar in the studied groups, with values ranging from 51 to 76 mg/dL.

After the first 2 weeks, the TG concentration in the n3 group was lower vs. initially and vs. the SD rats (*P <* 0.05). In the HFD and CA2 groups, TG were higher than initially and vs. the SD rats (*P <* 0.01) and the n3 rats (*P <* 0.001). In the HFD + n3 and CA1 groups, n-3 PUFAs did not offer significant protection against increased TG levels.

After 4 weeks, the HFD group continued to show increasing TG (*P <* 0.001 vs. 14 days previously). In the n3 group, the TG concentration was unchanged vs. 14 days previously but lower than in the SD rats (*P <* 0.05). In the HFD + n3 group, the TG concentration was higher than it had been 2 weeks previously (*P <* 0.001) but lower (*P <* 0.05) than in the HFD group, suggesting that n-3 PUFA protection against increased TG under HFD conditions is evident after a longer period than under SD conditions. In the CA1 and CA2 groups, the TG concentration was insignificantly different vs. the HFD and HFD + n3 groups and significantly higher than it had been 14 days previously (*P <* 0.01).

At 6 weeks, the HFD group continued to show a rising TG trend (*P <* 0.01 vs. 14 days previously). In the n3 group, TG concentrations were insignificantly lower vs. 14 days previously but significantly lower vs. the SD group, vs. initially (*P <* 0.01) and vs. 28 days previously (*P <* 0.05). The results suggest that the TG-lowering effect of n-3 PUFAs in individuals maintained on consistent diets depends on the supplementation time. In the HFD + n3 group, TG concentrations were lower than those in the HFD group (*P <* 0.05) and insignificantly higher than they had been 2 weeks previously. In the CA1 group, TG concentrations were lower vs. the HFD rats (*P <* 0.001), vs. the HFD + n3 group (*P <* 0.01) and vs. 2 weeks previously (*P <* 0.05). In the CA2 group, TG concentrations were lower vs. the HFD (*P <* 0.001) and CA1 groups (*P <* 0.05) but insignificantly lower vs. the HFD + n3 group and vs. 2 weeks previously, suggesting that reverting to a normal diet and adding PUFA as a dietary supplement after 4 weeks of exposure to HFD induces a dose-dependent TG-lowering effect.

### Morphological analysis

#### Aorta samples

**Fig. 2:**
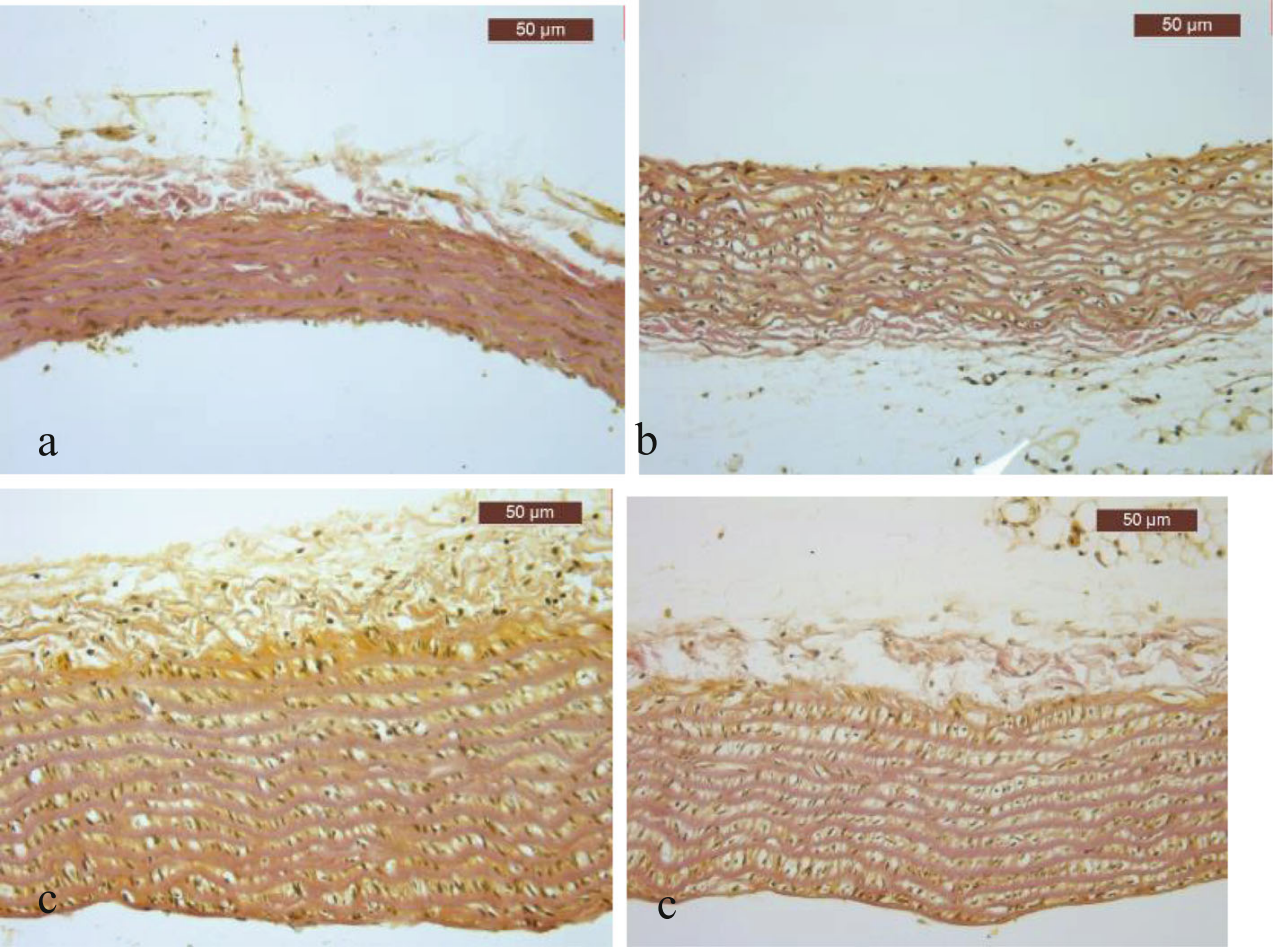
Histopathological aspects of aortic changes (van Gieson trichrome staining): **a** normal aorta after the first 14 days, all groups; **b** thickened aorta (12 collagen layers and vacuolated smooth muscle fibers) in an animal from the HFD group after 28 days; **c** thickened aorta in an animal from the HFD + n3 group after 28 days; **d** thickened aorta in an animal from the CA2 group at the end of the experiment

On gross examination under a magnifying glass, no changes in aortic wall thickness were observed. No adherent recent or organized thrombi were observed in the lumina of the aortic samples. After the first 14 days of exposure to different diets, the evaluation revealed insignificant changes in aortic wall thickness in the animals in the 6 groups. Thoracic aorta samples from all groups showed 6–8 layers of collagen in the media (Fig. 2a). In the SD and n3 groups, the number of layers remained constant during the three stages of the experiment. However, after exposure of the animals to different diets for 28 days, aortic wall thickness increased in the remaining study groups; increased numbers of collagen layers (8–12 layers) and hydropic changes in smooth muscle fibers were observed in the HFD + n3, CA1 and CA2 groups (Fig. 2b), and increased numbers of collagen layers (12–13 layers) were found in the HFD group (Fig. 2c).

Although the smooth muscle fibers continued to present hypertrophy or hydropic vacuolization at the end of the experiment (day 42), the number of collagen layers decreased to 8–10 in the HFD + n3 and CA1 groups, to 8–11 in the CA2 group, and to 10–12 in the HFD group (Fig. 2d). No classical atherosclerotic lesions (plaques) were observed in the examined aortic samples.

In all of the study group samples, the intimal layer was continuous; although the endothelial lining was focally interrupted, there was no fibrin network adherent to the basal membrane. No inflammatory infiltrate (neutrophils or lymphocytes) and no foamy cells (macrophages) were observed in the examined sections.

#### Liver

**Fig. 3:**
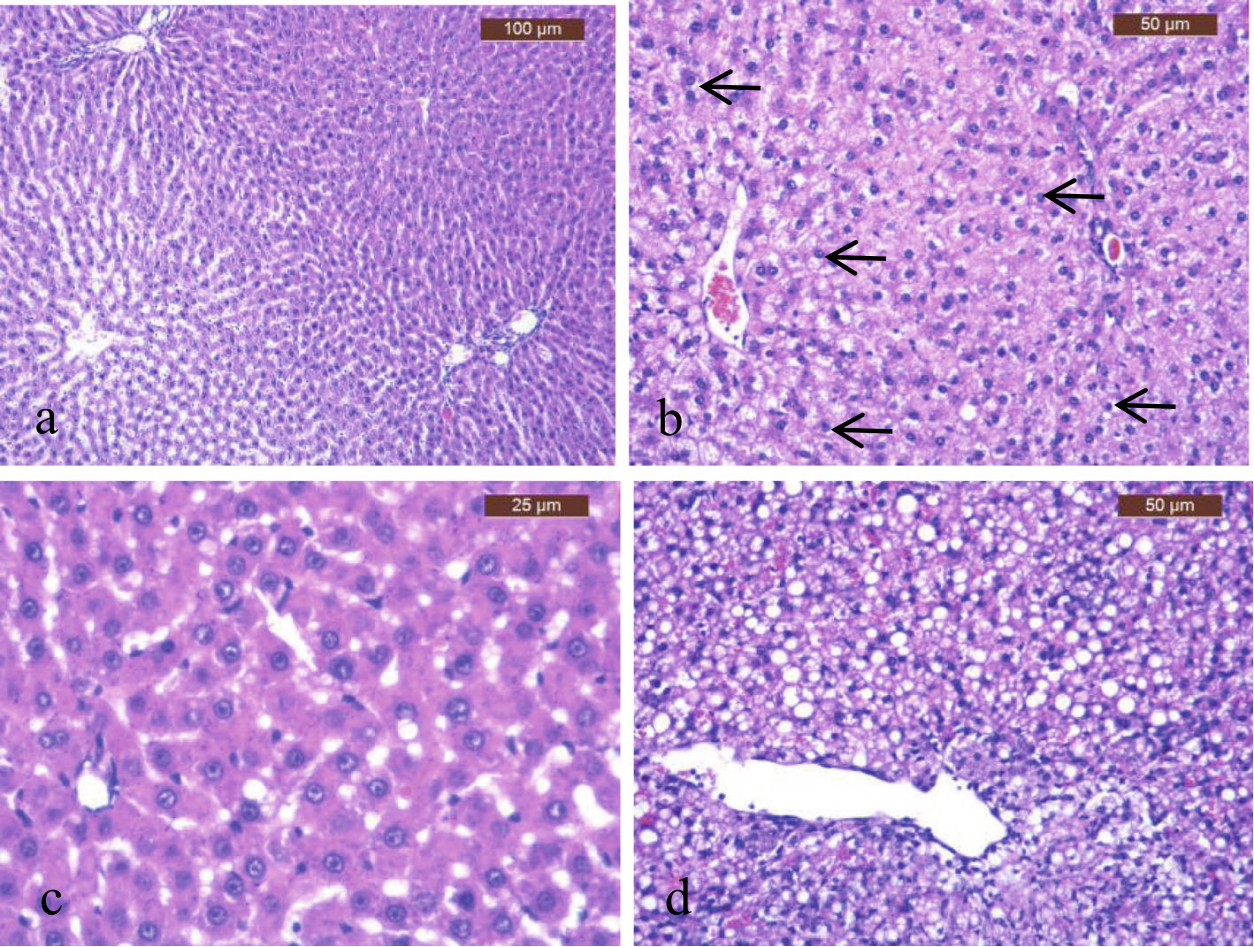
Histopathological aspects of liver changes (hematoxylin and eosin staining): **a** normal liver in the SD and n3 groups; **b** diffuse hydropic change *(arrows)* after 28 days in the HFD + n3 and CA1 groups; **c** grade I microvesicular fatty change in the HFD group at 28 days following the second stage; **d** stage III diffuse macrovesicular fatty change in the HFD group at 42 days

No morphological changes in liver tissue were observed in the animals in the SD and n3 groups during the experiment (Fig. 3a). At the end of the second phase of the experiment, hydropic changes in hepatocytes were observed in the HFD and CA2 groups (Fig. 3b), while in the HFD-n3 and CA1 groups, focal microvesicular fatty changes were observed in the hepatocytes surrounding the central vein (grade I fatty change, < 33% of hepatocytes) (Fig. 3c). After 42 days, grade III microvesicular fatty change was observed in the liver in the HFD + n3 and CA2 groups, and grade III macrovesicular fatty change (diffuse, > 80% of hepatocytes) was observed in the HFD group (Fig. 3d). Normal architecture without any fatty change in liver hepatocytes was observed in the CA1 group at the end of the experiment. Histopathological changes in the aorta and liver are synthetized in Table 4.

**Table 4:** Evolution of histopathological changes in the aorta and liver

Treatment	14 days		28 days		42 days	
	Aorta	Liver	Aorta	Liver	Aorta	Liver
**SD**	7–8 layers, smooth muscle/collagen	Normal	8 layers	Normal	7–8 layers	Normal
**n3**	7–8 layers, smooth muscle/collagen	Normal	7–8 layers	Normal	6–8 layers	Normal
**HFD**	7–8 layers, smooth muscle/collagen	Normal	12–13 layers, vacuolated smooth muscle fibers	Focal fatty change (periphery of central vein)	10–12 layers, hypertrophied and vacuolated smooth muscle fibers	Micro- and macrovesicular fatty change
**HFD + n3**	7–8 layers, smooth muscle/collagen	Normal	8–11 layers, hypertrophied and vacuolated smooth muscle fibers	Hydropic change	8–10 layers, hypertrophied smooth muscle fibers	Microvesicular fatty change
**CA1**	7–8 layers, smooth muscle/collagen	Normal	8–12 layers, hypertrophied and vacuolated smooth muscle fibers	Hydropic change	8–10 layers hypertrophied smooth muscle fibers	Normal
**CA2**	7–8 layers, smooth muscle/collagen	Normal	12 layers, hypertrophied and vacuolated smooth muscle fibers	Focal fatty change (periphery of central vein)	8–11 layers, hypertrophied and vacuolated smooth muscle fibers	Microvesicular fatty change

## Discussion

This study reveals a dose- and time-dependent positive effect of FO against WG, increased serum TG, TC, and LDL and decreased HDL, incipient atheromatosis and fatty change in rats under both normal and HFD conditions. Nevertheless, in HFD rats, FO protection, while evident, far from counteracted the consequences of the HFD. It has been shown here that the beneficial effect of FO is more evident when FO is administered at 50 mg/kg/day rather than at 25 mg/kg/day when added as a supplement to an SD for 2 weeks after 28 days on an HFD.

As expected, weight gain was observed in all groups during the 42 days on different dietary regimes, as the animals were 6 weeks old when the experiment began. However, WG was not equal among the groups: it was more evident in HFD rats that received no PUFA supplementation, while the addition of n-3 PUFAs to the HFD offered relative protection against excessive WG. Reduced WG due to n-3 PUFA administration was also observed in animals maintained under regular dietary conditions.

Many experimental studies have reported a protective effect of n-3 PUFAs against WG under both regular [28] and hyperlipidic diet conditions [29, 30]. However, some differing results have been reported. Addition of FO to an HFD for 13 weeks was shown to induce a slight (yet insignificant) WG increase [31], and Taouis et al. (2002) reported increased BW in the group fed an HFD rich in n-3 PUFAs compared to animals that received standard chow, but they used n-3 PUFAs at a considerably higher dose than the doses used in this study (addition of 6% peanut oil and 8% canola oil to the food) and not as a supplement, which significantly increased the animals’ caloric intake [32]. A diet rich in n-3 α-linolenic acid and containing 7% FO induced more WG than standard chow [33]. Using SD conditions, Wu et al. (2016) reported an insignificant BW-lowering effect of FO, but the dose they used was approximately 30 times higher than the dose used in the current experiment (1.5 g/kg/dose); thus, supplementary caloric intake may have been significant [34]. Addition of 2% cholesterol to the animals’ chow induced a decrease in food consumption after 4 to 8 weeks [35], while in this experiment the HFD induced more WG than regular food from weeks 1 to 6 without influencing food consumption.

In human studies, the effectiveness of FO in boosting weight loss has mainly been documented under balanced-diet conditions [36], but the results are somewhat controversial. Some studies report small changes in BW after FO supplementation, especially reduction of abdominal fat, but this is more evident when FO is combined with lifestyle changes [37]. Other studies report a lack of effectiveness of FO in promoting weight loss in men [38] and schoolchildren [39].

One hypothesis that has been advanced to explain the preventive role of n-3 PUFAs regarding WG relates to their influence on intestinal microbiota [40]. The role of n-3 PUFAs in improving serum lipid profiles has also been intensively studied.

In the current study, administration of ω-3 FA resulted in a greater decrease in LDL than in TC levels in the SD rats. In the HFD rats, n-3 PUFAs had a less evident LDL-lowering effect, while TC was more obviously decreased. Because FO generally increases HDL levels, it would be reasonable to assume that ω-3 FA supplementation under HFD conditions also decreases other types of cholesterol such as VLDL, IDL (intermediate-density lipoprotein) and chylomicrons (cholesterol-containing particles with a high TG content); possible changes in these cholesterol fractions were not measured in the current research. The HDL-increasing effect of adding FO to low-, moderate-, or high-cholesterol diets was shown by Surette et al. (1992) [41] in Syrian hamsters. On the other hand, Naik et al. (2018) [42] claimed a lack of effect of flaxseed oil (rich in ω-3 FAs) administration on HDL and LDL in rats fed a normal diet, while in HFD rats it decreased serum VLDL, LDL and TG. It is difficult to compare the doses used in the latter cited study to the doses used in the current research, but the administration period was twice as long in the experiment of Naik et al., 2018 than in this research. In animals fed a diet supplemented with 0.5% cholesterol, no TC-lowering effect of dietary n-3 PUFAs was observed [43]. It was reported that n-6 PUFAs lower TC more than n-3 PUFAs in hamsters maintained on a diet supplemented with 5% cholesterol [44].

An interesting situation was observed in the mixed-diet groups in the current study. In animals that consumed a hyperlipidic diet for 4 weeks followed by two weeks on the regular diet, neither dose of FO reversed the changes in cholesterol levels, with the exception of a slight increase in HDL (*P <* 0.05) in the CA1 group compared to the level observed 14 days previously.

FOs rich in n-3 FAs are known to reduce serum TG [45]. Such an effect was demonstrated under both SD and HFD conditions [42] but also under various other conditions: administration of 3 g/kg/day FO for 3 months decreased fasting plasma TG by 39% in a diabetes mellitus model [46]; after only 8 days of n-3 PUFA administration, serum and hepatic TG content decreased in rats treated with 0.5 mg/kg/day dexamethasone for 15 days but did not reach the control level [47], and a similar effect of dietary n-3 PUFAs was shown in rats with apical periodontitis, a condition in which serum TG is higher than in controls, but not in normal rats [48]. However, in the last-mentioned study, although the period of n-3 PUFA administration was similar to that in the present research, a 20% lower dose was used.

Reduction of serum TG by FO is mainly attributed to its n-3 PUFA content. There are several possible biochemical mechanisms, such as decreased very low-density lipoprotein cholesterol (VLDL) secretion in humans, and some animal research has suggested three general processes: reduced availability of FAs or other substrates (probably due to elevated b-oxidation), reduced hepatic delivery of free FAs and reduced liver FA synthesis; increased phospholipid synthesis; and decreased activity of TG-synthesizing enzymes (diacylglycerol acyltransferase or phosphatidic acid phosphohydrolase). However, it is difficult to ascertain whether such an effect primarily occurs in subjects with initially high TG levels and/or who are consuming high-fat diets or in individuals with normal lipid profiles. This study found a slight decrease in TG (*P <* 0.05) after 2 or 4 weeks of n-3 PUFA supplementation and a more evident decrease (*P <* 0.01) after 6 weeks in the SD rats; however, in the HFD groups, the TG-lowering effect of n-3 PUFAs was insignificant at 14 days but became evident (*P <* 0.05) at 4 and 6 weeks. In the CA1 group, the final 14 days of FO administration in conjunction with a normal diet were sufficient to lower TG to a level just slightly above that of the controls, but no such effect was seen in the CA2 group, which received only half of the CA1 FO dose. One might infer that FO is less efficient in lowering serum TG under HFD conditions than under regular lipid-content dietary conditions.

The current findings regarding serum TG are partially in agreement with the findings of Lin et al. (2005) [43], who reported that in rats and hamsters fed a cholesterol-free diet, dietary n-3 PUFAs lower plasma TG more effectively than n-6 PUFAs but that when diets are supplemented with 0.5% cholesterol, n-3 PUFA fails to induce such an effect.

The histological study revealed that aortic thickness decreased slightly or remained unchanged in the groups that received n-3 PUFAs (n3, HFD + n3, CA1 and CA2). Structural changes, especially in the aorta, develop more slowly than biochemical changes. Generally, it is known that an ω-3-rich diet has a protective role against atherogenesis. The mechanisms involved are related to improved serum lipid profiles and antioxidant activity, as shown in various epidemiological, clinical, and experimental studies [49–52]. Shen et al. (2018) [53] used an HFD 6-week model supplemented with a nitric oxide synthase inhibitor to induce atherogenic lipid modifications (increased TC, LDL, TG, and reduced HDL) and to facilitate the development of intracranial atherosclerotic stenosis in rats; they noted that under such conditions, supplementation with ω-3 FAs at a dose of 5 mg/kg/day resulted in an improvement in the blood lipid profile, with an absence of morphological changes. Some of the benefits associated with FA included reduced reactive oxygen species production and reduced nicotinamide adenine dinucleotide phosphate oxidase brain activity. In a similar experiment, the same authors (Shen et al., 2016) [54] showed that the luminal stenosis and intimal thickening of the middle cerebral artery induced by an HFD was attenuated by ω-3 FA administration, and the morphological improvement was accompanied by biochemical changes such as reduced levels of inflammatory markers [interleukin 6 (IL-6) and tumor necrosis factor α (TNF-α)], CD68, monocyte chemotactic protein (MCP-1), interferon-γ, brain expression of inducible nitric oxide synthase, and vascular cell adhesion molecule-1 (VCAM-1)]. PUFA intake has been linked to increased expression of adiponectin, a cytokine that reduces inflammation and decreases the risk of atherosclerosis [55].

Naik et al. (2018) [42] showed that administration of flaxseed (*Linum usitatissimum*, one of the richest sources of α-linolenic acid esters of plant sterols) to Wistar albino rats on an HFD improved not only the lipid profile but also the majority of the incipient histological atherosclerotic changes in the aorta. The treatment resulted in proliferation of endothelial cells and new vascular channel formation in the liver and between the cardiac muscle fibers. A study evaluating the effects of flaxseed oil demonstrated its efficacy in ameliorating atherosclerosis, optimizing overall lipid levels, and decreasing oxidative stress and inflammation [56]. A study in mice showed that fresh flaxseed oil can suppress hypercholesterolemic atherosclerosis. However, when the same oil was administered after being heated, it increased hepatic and plasma malondialdehyde (a potentially pro-atherogenic factor) and increased aortic wall thickness, lumen and diameter in mice on a regular diet [57].

The study also proved that FO provides relative protection against hepatic steatosis in a time- and dose-dependent fashion and probably through a mechanism that is related to the improvement in serum lipid profiles. This effect has been intensively studied, and the current findings are in agreement with the medical literature. For instance, in animal studies, it was reported that EPA exerted a protective effect against increased cholesterol and altered liver histology [58] and that flaxseed improved fatty liver changes in HFD rats [42]. Several human studies have also shown that administration of ω-3 FAs can alleviate liver morphological changes and lipid accumulation [59, 60]; one study even reported a reduction in liver fibrosis [61], but there are also research papers showing limited effectiveness of ω-3 FAs in non-alcoholic steatohepatitis [62].

Some data on the pathophysiology of hepatic lipid storage and the associated morphological changes are mentioned below as a background against which to consider the possible molecular mechanisms involved in the protective effect of n-3 PUFAs. Lipid accumulation initially involves insulin resistance and visceral obesity, which promote the synthesis of FAs from glucose and inhibit their β-oxidation. The excess FA leads to the synthesis of TG, which is stored in the liver, inducing high rates of mitochondrial β-oxidation and the consequent production of reactive oxygen species such as superoxide radicals (O_2_•-) and hydrogen peroxide (H_2_O_2_). High levels of such molecules, generated as a consequence of lipid peroxidation, inactivate the apoptotic caspase system and induce necrotic cell death. A decrease in antioxidant potential (superoxide dismutase activity and glutathione content) has also been demonstrated [63]. In a further stage, the progression to steatosis involves the activation of various transcription factors such as sterol regulatory element binding protein 1c (SREBP-1c), peroxisome proliferator-activated receptor γ (PPARγ) and carbohydrate responsive element-binding protein (ChREBP), which activate the expression of a series of genes essential for lipogenesis [64]. Other mechanisms include increased secretion by the adipose tissue of proinflammatory, fibrogenic and prothrombotic adipocytokines (IL-6, TNF-α) and reduced production of adiponectin, a potent anti-inflammatory, insulin-sensitizing adipocytokine. Inflammation is a component of the wound healing process and leads to the deposition of extracellular matrix and fibrosis [65].

N-3 PUFAs, especially EPA and DHA, regulate gene transcription factor activity and therefore act to control key hepatic lipid metabolic pathways. They potently activate PPARα, which in turn upregulates the expression of several genes involved in the stimulation of FA oxidation and downregulates the expression of genes encoding proinflammatory cytokines (TNF-α, IL-6). They also activate PPARγ, resulting in increased fat oxidation and improved insulin sensitivity. N-3 PUFAs decrease endogenous lipid production by inhibiting the expression and processing of SREBP-1, a stimulator of the transcription of several genes associated with lipogenesis and glycolysis that responds to increased glucose and insulin levels. N-3 PUFAs inhibit hepatic glycolysis and lipogenesis, suppress the activity of ChREB*P* (another regulator of glycolytic and lipogenic genes such as the L-pyruvate kinase and fatty acid synthase genes) [66] and increase the expression of adiponectin, a promoter of hepatic metabolic enhancement [57].

Certain aspects observed throughout the present study may be considered indicators of the limited effectiveness of FO in the case of high fat intake vs. a balanced dietary pattern. FO did not fully counteract the damaging effects of a cholesterol-rich diet, and its effect appeared weak even after a balanced diet was restored. For instance, when considering WG in developing animals, the n3 group exhibited lower weekly WG vs. controls between weeks 2 and 6 (SD conditions), but in the HFD rats the protection against excessive WG was restricted to weeks 3 to 5. FO supplementation for 14 days at 25 or 50 mg/kg/day in animals on an SD after 28 days of high fat intake did not reduce the trend to weight gain at weeks 5 and 6 in the CA1 and CA2 groups: there was no significant difference vs. the SD rats in the weekly WG percentage.

When considering the lipid profile, it could be seen that after the first 2 weeks of FO administration, TG concentrations decreased only under SD conditions (*P <* 0.05, SD vs. n3) and decreased insignificantly in the HFD rats. The TG-decreasing effect of n-3 PUFAs was less evident under HFD than under SD conditions at 42 days (n3 vs. SD, 0.001 < *P <* 0.01; HFD vs. HFD + n3, 0.1 < *P <* 0.05). The decrease in LDL level induced by n-3 PUFA administration was less evident under HFD than under SD conditions at 28 and 42 days. In the HFD rats, FO mainly decreased TC, while the decrease in the atherogenic LDL fraction, although significant, was less evident. An increase in HDL was only evident after 28 days of FO supplementation in the HFD rats, while in the SD rats it could be seen after only 14 days.

A course of 50 mg/kg/day of FO for 14 days in rats consuming an SD after 28 days on an HFD (CA1 group) just slightly decreased TG and increased HDL cholesterol vs. 14 days previously but had no effect on LDL or TC; half of the mentioned dose failed to induce any biochemical improvement in the CA2 group.

Diets lacking n-3 PUFAs (the HFD group) induced a progression of fatty changes in the liver from stage I to stage III. The observed significant alterations in liver morphology may represent a limiting factor for the possible benefit of n-3 PUFA supplementation, especially in the CA2 group.

The findings of the current study, which demonstrate the superior efficacy of n-3 PUFAs under SD vs. HFD conditions, are consistent with the findings reported in some of the above-cited research papers. In animals, ω-3 FAs evidently lower plasma TC and TG under SD conditions but not when diets are supplemented with 0.5% cholesterol [43]; supplementation of the diet with 5% cholesterol increased TC more when combined with n-3 PUFAs than when combined with n-6 PUFAs [44]. In humans, n-3 PUFAs are more effective in promoting weight loss when associated with a balanced diet [36, 37].

Currently, there is an increased tendency to consume foods rich in carbohydrates and fat but also an increasing trend in the use of dietary supplements such as vitamins and minerals (especially calcium, magnesium [67], and zinc [68]). In 2018, the global dietary supplements market was estimated to be worth 115.06 billion USD, and it is likely to expand. The global FO ω-3 market is forecast to reach 3057.03 million USD by 2024 [21]. North America is the most important consumer of such products [69]. Most of the available ω-3 supplements are FO products; the latter account for more than 75% of the total, followed by krill oil.

However, it is important to use supplements wisely, and increased awareness of their negative effects and limited effectiveness under certain conditions is advised.

Study strengths and limitations.

One of the main purposes of this study was to explore possible differences in the presumptive beneficial effects of dietary supplementation with n-3 PUFAs under standard, mixed and high-fat diet conditions. The animal model used involved continuous and intermittent exposure to HFD. The study design used two mixed diet models in animals in an attempt to simulate the diversity of human feeding patterns and to provide information on the presumed protective role of PUFAs on several relevant parameters under different conditions. The data related to the effect of n-3 PUFAs under mixed diet conditions that are similar to day-to-day human dietary patterns represent an element of novelty in the medical literature. There is still no standardized approach to testing the effects of PUFAs. It is therefore difficult to establish the extent of the protective role of FO [70]. The results obtained in this study showed that ω-3 has reduced effectiveness when its administration occurs during or follows a period of high lipid intake.

The study reproduced the situation in which n-3 PUFAs are administered as a dietary supplement rather than as a component of a so-called “healthy” diet.

The experimental research performed in this study is helpful to the extent that it offers a better understanding of certain mechanisms and helps predict the possible benefits of ω-3 PUFAs. However, the experiment does not completely mirror the complexity of pathogenic factors. Factors such as smoking, lifestyle, physical exercise, genetics, and age and their influence on lipid profile, atherosclerosis, and non-alcoholic liver disease were not taken into account. Certain aspects that influence feeding behavior (for instance, the impact of the high amount of fat in the HFD on taste perception and satiety) were not considered. The HFD was administered as a suspension added to the drinking water, whereas in humans high fat levels would normally be obtained from food, meaning that the greasy sensation would be more likely to be tempered by other different tastes. PUFAs were administered by oral gavage, a procedure that is unpleasant for the animal and might influence the amount of food and liquid consumed for a period of time afterward. Indeed, to reproduce the same stress conditions, a placebo was administered by the same procedure once daily to the subjects not receiving FO.

Other possible indirect effects of the applied treatments on feeding behavior (such as influences on mood) and on BW (such as influences on intestinal transit or locomotor activity, which was indeed very limited as the rats were maintained in individual cages) could not be evidenced.

Certain possibly relevant biochemical parameters were not assessed. These included VLDL, IDL, chylomicrons, fasting glycemia, insulin, glycated hemoglobin and circulating levels of n-3 PUFAs. VLDL assessment could have been specifically relevant because in the HFD rats, PUFAs decreased TC to a greater extent than LDL and increased HDL, while in SD rats they primarily lowered LDL rather than TC.

While atherosclerosis and hepatosteatosis are most often described in adult patients, this study was based on a rat model that was applied during the animals’ developmental period. In humans, atherosclerotic and liver modifications associated with HFD develop slowly over a long period of time (years), whereas the study period was limited to six weeks.

One additional limitation of the present study is that only two animals were euthanized for histopathological examination after each two-week period. Even so, the modifications were evident and characteristic and allowed pertinent conclusions.

## Conclusions

The current research shows that the metabolic, hepatic and cardiovascular benefits of consumption of ω-3-rich fish oil are more evident under balanced diet conditions than under HF diet conditions; fish oil cannot fully counteract the impairment induced by high fat intake, even when a standard diet is consumed following a period of excess fat consumption. These findings argue that more awareness should be promoted in the general population of the benefits of adopting a responsible attitude regarding a healthy diet and the relative lack of effectiveness of fish oil in preventing dyslipidemia and cardiovascular diseases if associated with high fat intake. It is necessary to distinguish between n-3 PUFAs administered as dietary supplements and a diet rich in natural sources of ω-3 FAs. From the clinical perspective, it is necessary to design experimental models in which combined feeding patterns are applied for a longer period of time and more closely replicate human feeding habits.

## Data Availability

The content of this manuscript has not been copyrighted or published previously. The content of this manuscript is not currently under consideration for publication elsewhere. The content of this manuscript will not be copyrighted, submitted, or published elsewhere while it is under consideration for acceptance by the Journal *Lipids in Health and Disease*. There are no directly related manuscripts or abstracts, published or unpublished, by any authors of this paper.

## Abbreviations

BW: Body weight
CA1: Combined alternative 1
CA2: Combined alternative 2
CCT: Standardized suspension containing 4% cholesterol, 1% cholic acid and 0.5% 2-thiouracil
ChREBP: Carbohydrate responsive element-binding protein
CVD: Cardiovascular diseases
DHA: Docosahexaenoic acid
DW: Distilled water
EPA: Eicosapentaenoic acid
FA: Fatty acids
FO: Fish oil
HDL: High-density lipoprotein cholesterol
HFD: High-fat diet
IL-6: Interleukin 6
LDL: Low-density lipoprotein cholesterol
PPARγ: Peroxisome proliferator-activated receptor γ
PUFAs: Polyunsaturated fatty acids
SD: Standard diet
SREBP-1c: Sterol regulatory element binding protein 1c
TC: Total cholesterol
TG: Triglyceride
TNF-α: Tumor necrosis factor α
VLDL: Very low-density lipoprotein cholesterol
WG: Weight gain
